# eHealth Literacy and Type 2 Diabetes Prevention Among At-Risk Populations: Mechanistic Systematic Review Using Theory-Driven Thematic Analysis

**DOI:** 10.2196/77788

**Published:** 2026-03-10

**Authors:** Jingyi Li, Arina Anis Azlan, Nurzihan Hassim, Yuan Wang, Ruina Guo

**Affiliations:** 1 Centre for Research in Media and Communication, Faculty of Social Sciences and Humanities, National University of Malaysia, UKM, Selangor, 43600, Malaysia, 60 123065261; 2 Shanxi Technology and Business University, Taiyuan, Shanxi, China; 3 Komunikasi Kesihatan (Healthcomm) - UKM Research Group, National University of Malaysia, Selangor, Malaysia; 4 Yuncheng University, Yuncheng, Shanxi, China

**Keywords:** eHealth literacy, digital health literacy, type 2 diabetes prevention, type 2 diabetes mellitus, self-management, chronic disease self-care

## Abstract

**Background:**

Type 2 diabetes (T2D) is emerging as a growing global public health crisis. Early and effective interventions can reduce T2D incidence among at-risk populations. Compared with traditional approaches, digital health technologies offer promising opportunities for prevention, with eHealth literacy (eHL) emerging as a critical determinant of digital prevention outcomes.

**Objective:**

This systematic review aims to synthesize and explain the pathways and mechanisms through which eHL supports T2D prevention among at-risk populations.

**Methods:**

We searched Scopus, Web of Science, and PubMed databases for English-language original research published between January 1, 2000, and August 14, 2025. Studies included were prevention research involving eHL engagement among populations at risk for T2D. Nonoriginal literature, such as editorials and abstracts, as well as research protocols, was excluded. The findings were synthesized using a thematic analysis approach, integrating the Theoretical Domains Framework with the eHL model. Two reviewers independently screened literature and extracted data, and discrepancies were resolved by a third reviewer. The Mixed Methods Appraisal Tool was used to assess risk of bias.

**Results:**

This review included 28 studies (n=13,100), mostly quantitative and published within the past decade, targeting people with prediabetes, prior gestational diabetes, and overweight/metabolic risk. Study quality was moderate to high (Mixed Methods Appraisal Tool 60%‐100%) with no high risk of bias. eHL supported prevention mainly through knowledge (28/28), behavioral regulation (16/28), social influences (15/28), environmental resources (12/28), and goals (11/28), while emotions, memory, attention, decision process, and beliefs about competence were rarely addressed. Health literacy (27/28), information literacy (20/28), and communicative eHL (20/28) were most common; critical eHL and media literacy were not addressed. Studies reported positive outcomes: high engagement, weight loss (≥5%), improved glycemic markers, and enhanced lifestyle behaviors.

**Conclusions:**

This is the first systematic exploration of eHL mechanism pathways in T2D prevention via theoretical mapping. We found interventions yield positive effects despite highly uneven mechanism application: extant research relies excessively on knowledge and behavioral pathways while underemphasizing emotional support, autonomy, and critical evaluation—factors linked to long-term adherence. We provide a mechanism-based framework and identify critical gaps, including the absence of focus on critical eHL and media literacy. This review is limited by substantial variation across studies that did not allow for meta-analysis and by the limited evidence base on eHL. Future interventions should explore and test emotional and autonomy support, information discernment training, and accessibility optimization in T2D prevention. These comprehensive, equity-focused intervention approaches will help ensure that eHL becomes a truly effective public health tool that benefits everyone, especially at-risk and vulnerable populations.

## Introduction

### Background

Type 2 diabetes (T2D) is becoming a growing public health crisis worldwide [1], accounting for 90% to 95% of all diabetes cases [2]. The global number of adults with diabetes is expected to surge from 589 million in 2024 to 853 million in 2050 [2], placing a huge burden on health care systems worldwide.

The development of T2D is driven by a variety of factors, among which unhealthy dietary habits and a sedentary lifestyle are the main triggers [3,4]. In addition, at-risk factors include overweight, obesity, insulin resistance, metabolic syndrome, dyslipidemia, and hypertension [5-7], genetics [8,9], nonalcoholic fatty liver disease, history of gestational diabetes [10], chronic stress [11], depression [12], sleep deprivation [13], etc.

Long-term exposure to or persistent presence of the aforementioned risk factors may increase the risk of developing dysglycemia and progressing to prediabetes. Prediabetes is an independent predictor of progression to T2D, and without timely intervention, individuals with prediabetes face a significantly increased risk of developing diabetes. Therefore, populations exhibiting one or more of these characteristics constitute the “at-risk population” for T2D [14].

Focusing prevention efforts on this at-risk population holds significant public health implications. Approximately 5%‐10% of individuals with prediabetes progress to diabetes annually, with a lifetime risk reaching up to 70% [15]. Notably, prediabetes and related risk states are not irreversible. Research indicates that individuals can regain normal glucose regulation through intensive lifestyle interventions [16], offering a critical window for preventive action. Furthermore, targeting prevention efforts toward at-risk populations demonstrates favorable cost-effectiveness in economic evaluations [17]. Despite the significant benefits of preventive interventions, the landmark Diabetes Prevention Program (DPP) study proved that intensive lifestyle intervention can reduce the risk of T2D by 58% in at-risk populations [18]. However, traditional face-to-face DPP programs face severe challenges, such as insufficient geographical and resource distribution limiting accessibility [19], high promotion cost [20], and low completion rate [21], which make it difficult to achieve comprehensive coverage. These limitations highlight the urgent need for innovative prevention models.

Digital health technologies offer promising solutions to overcome traditional barriers to prevention [22]. Interventions based on digital platforms have shown potential to improve access and reduce implementation costs [23]. However, with the rapid growth of web-based health information, there are significant differences in information quality and availability [24]. Some information may even mislead users and cause anxiety, which in turn has a negative impact on health decision-making [25].

In this digital context, eHealth literacy (eHL) has become a key factor in determining the effectiveness of digital prevention. Norman and Skinner [26] defined eHL as "the ability to seek, discover, understand, and evaluate health information from electronic resources, and to apply the knowledge gained to address or solve health problems” and divided it into 6 dimensions. In 2018, Paige et al [27] proposed a conceptual model in which eHL is described as an interpersonal skill and divided into 4 dimensions. Recent conceptual updates emphasize “the ability to use digital technologies to achieve health goals in effective, safe and beneficial ways” [28], reflecting the evolving nature of eHL in digital health settings.

Studies have confirmed that eHL significantly affects diabetes prevention behaviors [29]. High eHL can promote a healthy diet, regular exercise, and good sleep [30], and improve the use of the medical system [31]. Lack of eHL may hinder disease prevention and health management [32].

Despite the increasing importance of eHL, there is still a lack of mechanistic understanding and theory-driven synthesis regarding how eHL influences the prevention of T2D in at-risk populations. On the one hand, existing systematic reviews have generally studied the relationship between eHL and health behavior [33,34]; on the other hand, they have only examined the impact of digital health intervention or telemedicine on prevention, lacking attention to literacy. Or focus on disease management in patients with established disease [35-40].

This study aims to address this research gap by conducting a mechanistic systematic review using theory-driven thematic analysis. The main objective was to study the effect path of eHL on the prevention of T2D in people at risk of T2D and the participation ways of different dimensions. The findings are expected to provide evidence-based guidance to health educators, intervention designers, and policy makers to develop T2D prevention strategies tailored to at-risk populations.

### Research Questions

This mechanistic systematic review aimed to explore the pathways of eHL in the prevention of T2D among at-risk populations to fill the existing literature gap. Specifically, this study sought to answer the following research question (RQ):

- RQ1. What are the mechanistic actions of eHealth literacy in type 2 diabetes prevention among at-risk populations?
- RQ2. How do the dimensions of eHealth literacy facilitate type 2 diabetes prevention among at-risk populations?

## Methods

### Overview

The study protocol for this systematic review was registered with PROSPERO (registration number CRD42025630395 on January 16, 2025). This study was written and reported in accordance with the PRISMA (Preferred Reporting Items for Systematic Reviews and Meta-Analyses) guideline [41] (Checklist 1) and the PRISMA-S (Preferred Reporting Items for Systematic Reviews and Meta-Analyses–Search) [41] extension for reporting literature searches (Checklist 2).

### Data Resources and Search Strategy

Scopus, Web of Science (WOS), and PubMed were selected as the databases for this review. The literature search was conducted from January 1, 2000, to August 14, 2025. The year 2000 was chosen as the starting year because the concept of eHealth was formally proposed in 2001 [42]. Although the term eHL was proposed by Norman and Skinner in 2006 [26], earlier studies may have used related terms such as digital health literacy, health information literacy, etc, so a broad time frame was used to ensure the comprehensibility of the search.

In addition to database searching, we did not search trial or study registries; conduct any targeted searching or browsing of online or print resources; perform reference list screening or citation tracking; obtain additional studies or data by contacting authors, experts, or manufacturers; or use any other information sources or search methods. The search strategy was developed de novo, without adapting or reusing strategies from existing reviews, and no search update was performed (Multimedia Appendix 1). The strategy was peer-reviewed by an information scientist with extensive expertise in systematic review methods and database search strategy design.

Search terms were generated around the research topic. Two researchers (WY and RG) independently screened and identified relevant studies using retrieval strings based on the Population, Interest/Intervention, and Context model combined with Boolean logic. Disagreements were resolved by discussion and, if necessary, consensus was reached by consulting other investigators (AAA and ZZH). All keywords were searched in the database. A comprehensive list of search queries is presented in Table 1. Individual search strings for each database are shown in Checklist 2.

**Table 1.** Keywords search strings.

Concept	Keywords/search terms
eHealth	“eHealth” OR “e-health” OR “mHealth” OR “m-health” OR “mobile health” OR “digital health” OR “internet health” OR “telehealth” OR “telemedic*” OR “computer health” OR “computer-based health” OR “web health” OR “web-based health” OR “web based health” OR “online health” OR “online-based health” OR “online based health” OR “health” OR “media” OR “computer” OR “virtual” OR “intelligent” OR “technolog*” OR “Web 2.0” OR “web based” OR “online based” OR “phone*” OR “app*”
Literacy	“literac*” OR “comprehension” OR “skill*” OR “ability” OR “knowledge” OR “seek*“OR “find*” OR “search*” OR “understand*” OR “apprais*” OR “evaluat*” OR “assess*” OR “access*” OR “apply” OR “use*” OR “communication” OR “interaction” OR “efficacy” OR “engagement”
Type 2 diabetes prevention	“diabetes prevent*” OR “diabetes prevention” OR “type 2 diabetes prevent*” OR “type II diabetes prevent*” OR “T2D prevent*” OR “T2DM prevent*” OR “prediabetes prevent*” OR “non-insulin-dependent diabetes mellitus prevent*” OR “adult-onset diabetes prevent*” OR “NIDDM prevent*”
At-risk population	“at-risk” OR “high-risk” OR “risk population” OR “prediabetes” OR “overweight” OR “fat” OR “obesity” OR “sedentary behavi*” OR “sedentary lifestyle*” OR “physical inactiv*” OR “lack of exercise” OR “genetics” OR “family history” OR “gestational diabetes” OR “hypertens*” OR “dyslipid*” OR “insulin resistance” OR “acanthosis nigricans” OR “non-alcoholic fatty liver disease” OR “NAFLD” OR “metabolic syndrome” OR “chronic stress” OR “depression” OR “poor sleep” OR “western diet” OR “unhealthy diet” OR “suboptimal diet”

### Selection Criteria

Papers included in the review had to meet prespecified inclusion criteria. First, selected articles should be original research articles written in English and published between January 1, 2000, and August 14, 2025. Other literature types, such as reviews, editorials, conference papers, letters, briefings, reviews, book chapters, monographs, or research protocols, were excluded from this literature review. Second, people at risk for T2D. Patients included those with prediabetes, family history of diabetes, overweight or obesity, history of gestational diabetes, sedentary or lack of exercise, hypertension, dyslipidemia, insulin resistance, acanthosis nigricans, nonalcoholic fatty liver disease, metabolic syndrome, chronic stress, depression, inadequate sleep, or unhealthy diet. In addition, studies were excluded if they were (1) not specifically targeting T2D, but referring to diabetes or type 1 diabetes; (2) conducted via nonelectronic means such as text messages and phone calls; (3) only describing barriers or benefits of online prevention without explicitly mentioning prevention measures related to eHL; and (4) irrelevant to T2D prevention. Detailed inclusion and exclusion criteria are provided in Table 2.

**Table 2.** Inclusion and exclusion criteria.

Components	Inclusion	Exclusion
Population	Individuals at risk for type 2 diabetes, including those with prediabetes: a family history of diabetes; overweight or obesity; a history of gestational diabetes; a sedentary lifestyle or physical inactivity; hypertension; dyslipidemia; insulin resistance; acanthosis nigricans; nonalcoholic fatty liver disease; metabolic syndrome; chronic stress; depression; insufficient sleep; or an unhealthy diet.	Refers to diabetes without specifying people with type 2 diabetes, people with type 1 diabetes, and people without risk of type 2 diabetes.
Interest	Studies explicitly involving eHealth literacy in type 2 diabetes prevention among at-risk populations, either as an overall construct or through specific dimensions, using digital or online approaches.	Not specifically targeting type 2 diabetes, but referring to diabetes or type 1 diabetes. Nonelectronic means such as text messages and phone calls.
Context	Studies must report the impact of eHealth literacy on the prevention of type 2 diabetes among at-risk populations.	Only describe barriers or benefits of online prevention, without explicitly mentioning prevention related to eHealth literacy. Not associated with type 2 diabetes prevention.
Timeline	January 1, 2000, to August 14, 2025	Before 2000.
Article type	Primary research journal articles on quantitative research, qualitative research, and mixed methods research	Reviews, editorials, conference papers, letters, notes, commentaries, book chapters, books, or study protocols.
Language	English	Non-English.

### Systematic Review Process

All papers retrieved from WOS, Scopus, and PubMed databases were imported into Rayyan Citation Manager [43], and duplicates were removed. Two authors (WY and RG) independently screened studies according to prespecified inclusion and exclusion criteria. Studies that may meet the criteria will be screened for full text for a final decision on inclusion in our review. Any inconsistencies were resolved through discussion and consensus among the reviewers. If consensus was not reached, a third reviewer (AAA) made the final decision.

### Risk of Bias Assessment and Reporting Bias Assessment

The risk of bias of all included studies was independently assessed by 2 reviewers (WY and RG) and cross-validated by each other. Any disagreements were resolved by discussion with a third evaluator (AAA). The Mixed Methods Appraisal Tool (MMAT, version 2018) was used to assess the quality of the eligible studies. This tool is primarily used to assess the quality of mixed methods studies and also includes the assessment of qualitative and quantitative methods. MMAT included 2 screening questions for each study and then 5 questions for each design, depending on the category of study design. Each question was rated as “no,” “yes,” and “unable to judge.” The risk of bias was determined for each study based on the percentage of questions answered “yes,” with scores ranging from 0% to 100% [44]. Prior to conducting the assessment, reviewers completed the official MMAT online training tutorial developed by Pace et al [45] to ensure consistent and informed use of the tool.

### Theoretical Underpinnings

This systematic review uses the Theoretical Domains Framework (TDF) and the eHL model as its theoretical foundation for constructing a coding framework, organizing and interpreting evidence, and identifying behavioral mechanisms and literacy dimensions that are adequately covered versus relatively underrepresented in current intervention designs during the thematic synthesis process.

The TDF encompasses 14 domains, including knowledge, skills, social or professional roles and identities, beliefs about competence, optimism, reinforcement, intentions, goals, memory, attention and decision processes, environmental background and resources, social influences, and emotions and behavioral regulation [46]. This framework provides a systematic and actionable theoretical classification to elucidate the potential mechanisms through which eHL may influence diabetes prevention-related behaviors, further pointing to intervention targets for optimization.

To comprehensively describe the dimensional coverage and mechanistic actions of eHL in the included studies, this research uses 2 complementary eHL models to balance breadth and depth. At the breadth level, the Norman and Skinner framework conceptualizes eHL as 6 foundational literacies (traditional literacy, health literacy, information literacy, scientific literacy, computer literacy, and media literacy) [26], facilitating cross-comparisons of eHL dimensional coverage across studies. At the depth level, the interactive framework of Paige et al [27] refines eHL into functional eHL, communicative eHL, critical eHL, and translational eHL, emphasizing interaction, comprehension, and application transfer within specific digital health contexts. The integration of these models enables this review to provide a more systematic interpretation and comparative analysis of eHL’s multidimensional mechanisms within the context of T2D prevention.

### Data Extraction and Analysis

To gain a comprehensive understanding of the essential characteristics and trends of the included studies, we developed a set of standardized data extraction tables for summarizing key study characteristics. The table collected detailed information from the included literature, including author, year of publication, country/region, age of participants, study methods, and main intervention outcomes.

In addition, we use thematic analysis as the main method to identify patterns or themes in qualitative data [47]. It is widely regarded as one of the most appropriate methods for integrating mixed study designs [48]. Thematic analysis is a descriptive strategy that enables flexible reduction of data and can be used in combination with other data analysis techniques [49].

Therefore, we conducted a thematic analysis to identify common themes that emerged in the data. These themes were derived from the objectives of this review.

First, addressing RQ 1, two reviewers (WY and RG) classified relevant findings by mapping intervention components and reported implementation strategies to the 14 domains of the TDF [46], to summarize which behavior change mechanisms were reflected across studies.

Second, addressing RQ 2, we examined how different dimensions of eHL contribute to the prevention process by mapping eHL-related content from included studies onto 2 complementary eHL models [26,27].

In addition, we used the same thematic analysis method to generalize the intervention outcomes reported by the studies into weight change, diabetes-related measures, behavior change, self-efficacy, and engagement and acceptability.

For studies independently reviewed by 2 researchers (WY and RG), if consensus cannot be reached, the final decision will be made by a third reviewer (AAA).

## Results

### Study Screen

#### Overview

In accordance with PRISMA 2020 reporting guidelines, we conducted a comprehensive search on WOS, Scopus, and PubMed to find 5409 articles published in English. During the initial title and abstract screening process, we identified and removed 2593 duplicate records, resulting in 2816 articles progressing to the next stage.

**Figure 1.**
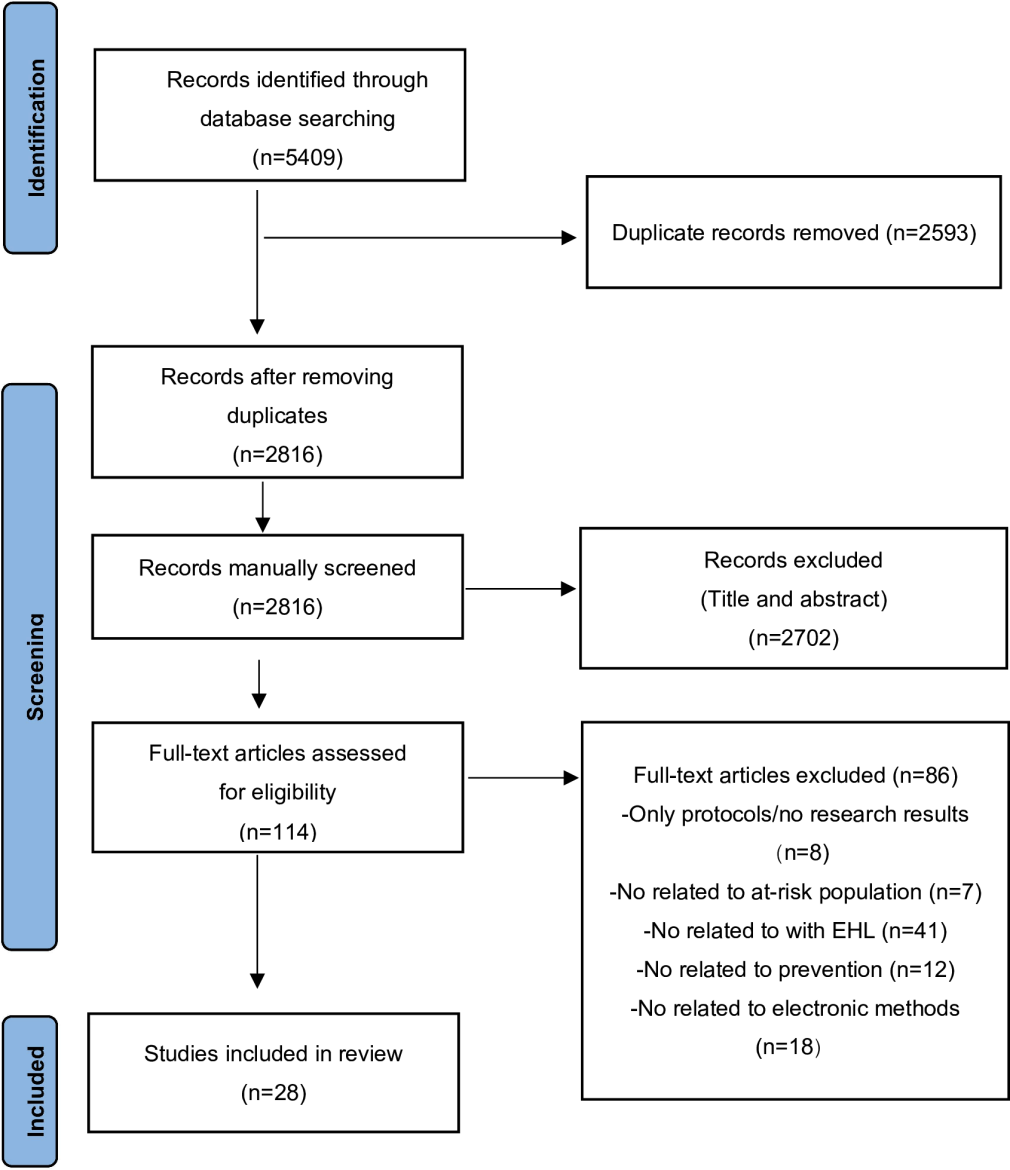
Flowchart of systematic review search results. eHL: eHealth literacy.

After further review of titles and abstracts, 2702 articles were excluded due to lack of relevance to eHL or T2D prevention in at-risk populations, leaving 114 articles for full-text screening. Of these, 86 articles were subsequently excluded. Ultimately, 28 studies were included. The PRISMA screening flow chart is shown in Figure 1 for details.

#### Description of Studies

This review includes 28 studies that met the inclusion criteria, with their characteristics detailed in Table 3.

The majority of studies were conducted within the last decade (27/28), with a particular focus on 2019, 2022, and 2023 [50-62]. One such study was conducted in 2010 [63].

A total of 86% (24/28) of the studies were from the United States. In addition, there was one study from Canada (n=1), one study from Malaysia (n=1), one study from Taiwan, China (n=1), and one study from Singapore (n=1).

In terms of population at-risk, 23 studies focused on people with prediabetes [50-72], 3 studies focused on women with a history of gestational diabetes [59,63,73], and 10 studies examined individuals who were overweight, obese, had excessive BMI, or had metabolic syndrome [53,56,58,59,67-69,71,74,75]. In addition, one study included individuals who had previously given birth to a baby weighing more than 9 lb (about 3.7 kg) and had hypertension or dyslipidemia [63].

Seven studies [51,53,54,58,60,64,76] specifically addressed special populations, including female veterans [64], low-income individuals enrolled in Medicaid [51], self-identified as Asian [53], adolescents [54], older at-risk individuals [58,76], and Chinese Americans [60].

A total of 18 studies used web-based DPP interventions [51,53,55,57-60,62-64,66,67,69,70,72,74,76,77], and one study specifically recruited a population not previously enrolled in a DPP program [52].

A total of 28 intervention studies were ultimately included, encompassing 13,100 participants. Intervention durations ranged from 12 weeks to 3 years, with participant ages spanning adolescents to older adults (approximately 14.6 to 69.2 y).

**Table 3.** Characteristics and key findings of included studies on type 2 diabetes prevention among at-risk populations.

Study	Country	Study design	Sample size	Characteristics of at-risk populations	Average age (years)	Gender	Intervention delivery modality	Time frame	Key findings
Vadheim et al (2010) [63]	United States	Quantitative nonrandomized	29	A history of prediabetes, gestational diabetes mellitus (GDM), or delivery of a baby weighing more than 9 pounds, hypertension, or dyslipidemia	51.4	81.4% (22/27) women	Remote videoconference	16 weeks	A total of 88% (14/16) of participants completed the 16-week program; More than 40% (12/29) of participants achieved the 7% weight loss goal.
Moin et al (2015) [64]	United States	Qualitative study	15	Female veterans with prediabetes	56.8	100% (15/15) women	Online virtual platform, pedometer, tracker, wireless scale	16 weeks	Perception: the plan was well aligned with perceived health needs; The program is convenient; Integration of projects into daily life; A sense of responsibility; Hate records; Prefer traditional offline; Difficult to understand; Mean weight loss was 5.24%.
Sepah et al (2015) [74]	United States	Quantitative nonrandomized	220	BMI>24 kg/m^2^ over 18 years of age	43.6	17.3% (38/220) men	Online web platform, digital tracking tools (wireless scale and pedometer tracking)	2 years	Users had significant reductions in body weight and HbA_1c_^a^ that were sustained 2 years later.
Block et al (2015) [65]	United States	RCT^b^	339	Prediabetes	55	68.7% (233/339) men	Alive-PD app, personalized website, and interactive email delivery	6 months	Reductions in HbA_1c_, fasting glucose, and body weight were significantly better in the intervention group than in the control group. Health behaviors and lifestyle improved significantly, and program participation was high.
Sepah et al (2017) [66]	United States	Quantitative nonrandomized	220	Prediabetes	—^c^	82.7% (182/220) women	An Internet-connected desktop computer or mobile device	3 years	There was a significant decrease in body weight and glycated hemoglobin at week 16, year 1, and year 2. At the third year, there was a significant reduction in body weight and HbA_1c_.
Stein and Brooks (2017) [75]	United States	Quantitative nonrandomized	70	Overweight and obese adults	47	74.5% (35/47) women	Artificial Intelligence Health Coaching app (HCAI app)	15 weeks	A total of 75.7% (53/70) of the users lost weight in this plan, and the percentage of healthy diets increased by 31%. The response rate of the user trust survey is 100%.
Michaelides et al (2018) [67]	United States	Quantitative nonrandomized	59	Overweight or obese adults with prediabetes	51	81% (48/59) women	The Noom application	65 weeks	Weight and BMI were significantly reduced, with 53% (25/47) of the completion group and 66% (21/32) of the maintenance group achieving ≥5% weight loss.
Everett et al (2018) [68]	United States	Quantitative nonrandomized	55	Adults with prediabetes and BMI between 24 and 40 kg/m^2^	55	60% (33/55) women	Noom application, Sweetch application, and Digital Weight Scales (DBWS)	3 months	The retention rate and acceptance of the study were relatively high. Participants lost 1.6 kilograms in weight, their BMI decreased by 0.6 kilograms/m^2^, their waist circumference reduced by 1.4 centimeters, and their physical activity significantly increased. Fasting blood glucose showed no significant change compared with the baseline.
Castro Sweet et al (2018) [69]	United States	Quantitative nonrandomized	501	Have prediabetes or metabolic syndrome	68.8	64% women^d^	Connected devices, wireless scales	12 months	The participants lost an average of 6.5% in weight over 16 weeks. At the 6th month, the weight decreased by 8.0%, and at the 12th month, it decreased by 7.5%. The participants' blood sugar control was improved and their total cholesterol was reduced. Participants' self-reported improvements in well-being, depression, and self-care.
Srivastava et al (2019) [50]	United States	Quantitative nonrandomized	10	Prediabetes	56.1	70% (7/10) women	Cloud-based type 2 diabetes prevention module	6 months	The success rate of the module is approximately 60%. The average weight loss of users is approximately 9.0%, and their weekly physical activity time is 192 minutes.
Kim et al (2019) [51]	United States	Quantitative nonrandomized	227	Low-income people with prediabetes enrolled in Medicaid	48.2	81.3% women^d^	Online digital platforms	12 months	The intervention group had an average weight loss of 4.4% and an average BMI change of –1.6 over 12 months.
Griauzde et al (2019) [52]	United States	Mixed methods	69	People with prediabetes who did not participate in DPP^e^	51.7	64% (44/69) women	Apps, Fitbit devices	12 weeks	8 out of 13 respondents liked some features of the app, and 13 out of 13 respondents reported changes in health behaviors.
Alwashmi et al (2019) [53]	United States	Quantitative nonrandomized	273	Prediabetes, BMI>25 kg/m^2^, or self-identified as Asian	54	70.3% (192/273) women	Interactive mobile computing (eg, smartphone applications), wearable tracking devices (eg, activity trackers), remote health monitoring hardware (eg, electronic scales)	4 months	The average weight loss was 6.5%, the average BMI decreased by 1.9 kg/m^2^, the frequency of exercise increased by 1.7 days per week, and the monthly absenteeism rate decreased by nearly half a day.
Toro-Ramos et al (2020) [70]	United States	RCT	202	Prediabetes	56.6	71.3% (144/202) women	The Noom application	12 months	The changes in body weight and BMI in the intervention group were significantly reduced, but the effect on HbA_1c_ was not significant in the intention-to-treat analysis.
Ferrara et al (2020) [77]	United States	RCT	158	People with a risk of more than 9 points were screened by the tool	52.2	72.8% (115/158) women	Canary health online platform	12 months	The online group achieved significant weight loss within 6 months, with an average weight change of –1.97 kilograms. 12 months –1.27 kilograms; At 6 months and 12 months, the average total calorie intake, dietary fat intake, and saturated fat intake of the online group all decreased significantly. However, it did not lead to significant changes in physical activity.
Ekezie et al (2021) [73]	United States	Qualitative study	23	Women with previous Gestational Diabetes Melilitus	—	100% (23/23) women	Baby steps app	11 months	Changes in lifestyle; Increasing physical activity and steps; Promoting competition and communication; Increase motivation; Bring in peer support.
Islam et al (2022) [54]	United States	Qualitative study	14	Adolescents with prediabetes	14.6	36% (5/14) women	Zoom	12 weeks	Participants indicated that the online adolescent health education program was beneficial and applicable to their lives, and gave them a deeper understanding of healthy eating and active lifestyle.
Katula et al (2022) [55]	United States	RCT	599	Prediabetes	55.4	61.4% (368/599) women	Internet-connected device (laptop, tablet, or smartphone)	12 months	HbA_1c_, body weight, and cholesterol/high-HDL^f^ ratio decreased significantly. Reductions in HbA_1c_ and body weight were greater in the older participants aged ≥65 years.
Lim et al (2022) [56]	Singapore	RCT	148	Adults with prediabetes and BMI ≥23 kg/m^2^	53.1	39.9% (59/148) women	nBuddy Diabetes app	6 months	The intervention group was 2.1 times more likely to achieve euglycemia (HbA_1c_<5.7%) than the control group. Within-group improvements in systolic and diastolic blood pressure, total cholesterol, and low-density lipoprotein (LDL) cholesterol were significant in the intervention group. The intervention group had significantly greater reductions in daily intake of total calories, carbohydrates, total fat, saturated fat, and sugar than the control group.
Batten et al (2022) [57]	Canada	Quantitative nonrandomized	1095	Prediabetes	53.6	67.7% (741/1095) women	Smartphone applications, wireless scales, wearable tracking devices	12 months	After at least 9 months of participation, the average weight loss was 11.4 pounds, with a rate of 5.5%. Mean weekly physical activity increased to 132.9 minutes.
Fitzpatrick et al (2022) [58]	United States	Quantitative nonrandomized	3904	65-75 years with prediabetes and obesity	69.2	55.2% (2155/3904) women	Electronic patient portal, wireless scale, and pedometer	24 months	The average weight loss was 8.6 pounds, and participation was high.
Collins et al (2023) [59]	United States	Quantitative nonrandomized	2390	Adults with BMI >25 and a history of gestational diabetes or prediabetes	54.9	89.2% (2132/2389) women	Video conferencing, secure online portals	12 months	HbA_1c_ monitoring decreased significantly, and those who completed the program lost an average weight of 5.66%. Participants showed statistically significant changes in their self-reported confidence in their ability to perform all 18 health promotion behaviors.
Yeh et al (2023) [60]	United States	Mixed methods	13	Chinese Americans with prediabetes	66	85% (11/13) women	Facebook groups, Qualtrics links	12 months	The completion rate was high. The completion rate of the maintenance phase decreased slightly; the weekly modules were generally considered to be clear and easy to understand. All of the participants (100%) liked the online DPP, and the overall satisfaction of the participants was high. Body weight decreased significantly at 2, 3, 4, 8, and 10 months.
Zahedani et al (2023) [61]	United States	Quantitative nonrandomized	2217	Prediabetes	49	51% women^d^	January AI app, wearing CGM (Freestyle Libre, Abbott), heart rate monitor (Apple Watch or Fitbit)	About 3 months	The GMI^g^ was significantly reduced, and the number of prediabetic hyperglycemia events was the largest reduction. Prediabetic individuals lost an average of 2.5 pounds, roughly doubled their physical activity, and increased their heart rate by 6.3 minutes/day; Calorie, carbohydrate, sugar, and saturated fat intakes all decreased, whereas protein, total fat, and dietary fiber intakes increased.
Chung et al (2023) [62]	Taiwan, China	RCT	121	Prediabetes	58.1	52.9% (64/121) women	TCM mHealth app	About 4 months	The TCM mobile health app intervention was superior to the control group in blood glucose control (HbA_1c_ reduction), body constitution (Yang deficiency, phlegm stasis improvement), body energy improvement, health-related quality of life (PCS, MCS), and BMI reduction.
Gerber et al (2024) [76]	United States	RCT	20	Older people with excessive BMI	65.4	45% (9/20) women	Zoom, Fitbit Inspire 2 /Fitbit Aria Air, Fitbit App	6 months	Meeting participation was high, with 74% calorie intake compliance; Body weight and BMI changed significantly. The intervention group achieved an 8.4% reduction in body weight at 6 months while BMI decreased by 2.3 units at 3 months.
Ng et al (2025) [71]	Malaysia	RCT	82	Overweight/obese prediabetic patients	49.8	53.7% (44/82) women	Blood glucose meters and wearable fitness trackers, PRIME app, WhatsApp	6 months	The dietary quality score, vegetable score, and sodium score were significantly improved in the intervention group. The intervention group showed beneficial changes in refined grains, vegetable consumption, and sodium intake.
Audet et al (2025) [72]	United States	Qualitative study	27	Prediabetes	56	89% (24/27) women	HP-DPP program (Zoom+REDCap app)	12 months	The participants' knowledge, motivation, and skills were improved.

a HbA_1c_: glycosylated hemoglobin A_1c_.

b RCT: randomized controlled trial.

c Not available.

d N values are not available.

e DPP: Diabetes Prevention Program.

f HDL: density lipoprotein cholesterol.

g GMI: glucose management index.

Most studies were quantitative (n=22), including randomized controlled trials (n=8) [55,56,62,65,70,71,76,77] and nonrandomized quantitative studies (n=14). These include retrospective studies [50,53,57], interventional studies [51,61], observational studies [58,59,67-69,75], experimental studies [66,74], and pilot studies [63].

In addition to this, 2 studies used a mixed methods design [52,60] and another 4 studies were qualitative [54,64,72,73].

Among the 28 included studies, the delivery modalities used to deliver T2D prevention interventions were categorized into 4 groups. The first 10 involved apps for diabetes prevention [53,56,61,62,65,67,70,72,73,75] and the second 13 involved the use of web-based platforms or websites [50,51,53,55,58-60,64-66,69,74,77]. In addition, 11 trials used wearable devices or digital tracking tools such as a wireless scale [52,53,57,58,61,64,68,69,72,74,76]. Six studies used video conferencing or social software such as Zoom and Facebook [54,59,60,63,72,76].

#### Bias Reporting of the Eligible Studies

The MMAT was used to assess the risk of bias across the 28 included studies, with none identified as having a high risk of bias. The overall methodological quality was rated as moderate to high, with MMAT scores ranging from 60% to 100% across the studies (Table 4).

**Table 4.** Quality assessment.

Study	Research design	Q1	Q2	Q3	Q4	Q5	Quality	Inclusion in the review
Vadheim et al (2010) [63]	QN (NR)^a^	1	1	1	0	1	80%	✓
Moin et al (2015) [64]	QA^b^	1	1	1	1	1	100%	✓
Sepah et al (2015) [74]	QN (NR)	1	1	0	0	1	60%	✓
Block et al (2015) [65]	QN (RC)^c^	1	1	1	1	1	100%	✓
Sepah et al (2017) [66]	QN (NR)	1	1	1	0	1	80%	✓
Stein and Brooks (2017) [75]	QN (NR)	1	1	1	0	1	80%	✓
Michaelides et al (2018) [67]	QN (NR)	1	1	1	0	1	80%	✓
Everett et al (2018) [68]	QN (NR)	1	1	1	0	1	80%	✓
Castro Sweet et al (2018) [69]	QN (NR)	1	1	1	0	1	80%	✓
Srivastava et al (2019) [50]	QN (NR)	1	1	0	0	1	60%	✓
Kim et al (2019) [51]	QN (NR)	1	1	1	1	1	100%	✓
Griauzde et al (2019) [52]	MX	1	1	1	0	1	80%	✓
Alwashmi et al (2019) [53]	QN (NR)	1	1	1	0	1	80%	✓
Toro-Ramos et al (2020) [70]	QN (RC)	1	1	1	1	1	100%	✓
Ferrara et al (2020) [77]	QN (RC)	1	1	0	1	1	80%	✓
Ekezie et al (2021) [73]	QA	1	1	1	1	1	100%	✓
Islam et al (2022) [54]	QA	1	1	1	1	1	100%	✓
Katula et al (2022) [55]	QN (RC)	0	1	1	1	1	80%	✓
Lim et al (2022) [56]	QN (RC)	1	1	1	1	1	100%	✓
Batten et al (2022) [57]	QN (NR)	1	1	1	0	1	80%	✓
Fitzpatrick et al (2022) [58]	QN (NR)	1	1	1	1	1	100%	✓
Collins et al (2023) [59]	QN (NR)	1	1	0	1	1	80%	✓
Yeh et al (2023) [60]	MX^d^	1	1	1	0	1	80%	✓
Zahedani et al (2023) [61]	QN (NR)	1	1	1	0	1	80%	✓
Chung et al (2023) [62]	QN (RC)	1	1	1	1	1	100%	✓
Gerber et al (2024) [76]	QN (RC)	1	1	1	1	1	100%	✓
Ng et al (2025) [71]	QN (RC)	0	1	1	0	1	60%	✓
Audet et al (2025) [72]	QA	1	1	1	1	1	100%	✓

a QN (NR): Quantitative nonrandomized.

b QA: Qualitative assessment.

c QN (RC): Quantitative randomized controlled trials.

d MX: Mixed Method.

### The Mechanism of Action of eHL

#### Knowledge

**Figure 2.**
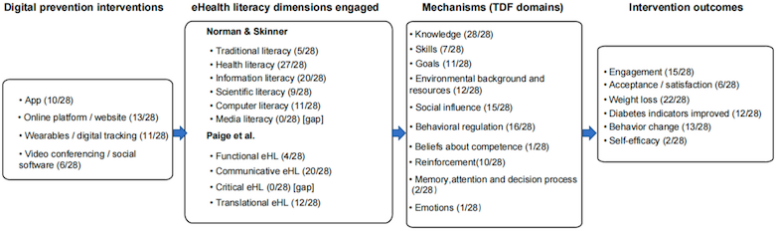
Mechanistic pathways of eHL in type 2 diabetes prevention among at-risk populations [26,27]. eHL: eHealth literacy.

Figure 2 illustrates the pathways and mechanisms through which eHL supports T2D prevention among at-risk populations.

##### Overview

All 28 included studies (100%) addressed the mechanistic actions of health literacy in the prevention of T2D. The content covered disease cognition, nutrition and diet, physical activity, and standardized DPP courses. These interventions impact diabetes prevention through 3 pathways as follows.

##### Information Acquisition and Understanding—Disease Knowledge (n=3)

Such as prediabetes and strategies to prevent progression of T2D [52], understanding prediabetes [62], and the pathophysiology and complications of prediabetes [71], the acquisition of disease knowledge builds the knowledge base for prevention.

##### Behavior Guidance—Diet and Exercise Knowledge (n=8)

Nutrition and diet knowledge was the most common content, Content covering topics such as dietary sources of saturated fats and sugars, portion control [65], weight management, diabetes prevention, healthy meal planning, the relationship between carbohydrates and glycemic response [56], balanced meals following the US Department of Agriculture’s My Plate program [57,58], diet [60], reducing foods that raise blood glucose, restricting calories, and increasing fiber intake [61], the Dietary Approaches to Stop Hypertension diet [62], practical nutrition advice for people with prediabetes, including energy balance, portion control, healthy eating based on local foods, glycemic index, fiber intake, variety of fat and protein foods, alcohol intake, etc [71].

Physical activity knowledge (n=6) included teaching classes related to physical activity, such as aerobic exercise, strength training, yoga, dance, pilates, water aerobics, etc [63], physical exercise guidelines [65], educational videos on physical activity [56], benefits and recommendations for increasing activity [61,62] of physical activity [71]. The guidance of diet and exercise knowledge promotes prevention from information to practice.

##### Digital Intervention Program—Systematizing Knowledge (n=18)

A total of 18 studies [51,53,55,57-60,62-64,66,67,69,70,72,74,76,77] were based on the DPP or its digital adaptations, including 2 from the Prevent project [64,74] and 5 from the Omada project [55,57,58,66,69]. And 11 derived from DPP and its derivative programs [51,53,59,60,62,63,67,70,72,76,77], whose curricula deliver knowledge through integrated multimodal digital channels.

### Skills

#### Overview

Seven studies [52,54,56,62,67,70,77] reported that eHL promotes prevention through skill building.

#### Digital Tool Operation Skills (n=6)

eHL is reflected in the ability of participants to learn and master digital intervention tools. Six studies provided training in tool use [52,56,62,67,70,77]. These included learning how to operate the Noom App and interacting with a web-based coach [67,70], receiving instructions for using the App and Fitbit devices [52], learning how to use the online platform through face-to-face group coaching, goal setting, and behavioral techniques for self-monitoring diet, physical activity, and weight using the online platform [77], and downloading and familiarizing yourself with mobile health applications [56,62].

#### Health Behavior–Related Skills (n=1)

One study [54] reported participants learning practical health management skills through a digital platform, including reading nutrition labels, making plate plans, and controlling portion sizes.

### Goals

#### Overview

A total of 11 studies [54,56-58,62,65,68-71,73,75] provide the function of goal setting in diabetes prevention. There are 3 main patterns of goal setting in research.

#### Algorithm-Driven Personalized Target Recommendation (n=3)

Three studies used algorithms to generate personalized goals for participants [62,65,68]. In a high-quality study, the Alive-PD system recommended weekly that participants choose one eating habit goal and one physical activity goal from among the suggested goals. In addition to long-term goal setting, individually relevant small-step goals were recommended weekly according to the participant. For example, participants who consumed more than the recommended level of added sugars were provided with weekly targets based on their reported sources of added sugars. The target-recommendation algorithm adapts to the participant’s previous performance. They were also prompted on a weekly basis to report on the completion of goals in the previous week. The system tracks the number and type of goals that respondents successfully achieve [65]. In another study, Sweetch used advanced algorithms to provide personalized, contextualized, immediate, and timely advice to guide users toward recommended activity, weight loss, and dietary goals in a way that aligns with the user’s actual lifestyle. In another high-quality study, the Sweetch technique was able to learn which types of messages led to better compliance for specific users in specific contexts. Breaking down the weekly 150-minute physical training goal into small pieces throughout the day makes the goal easier to achieve. These snippets will continue to be personalized for each user. Personalized goals are constantly adjusted according to the actual behavior of the user to best fit the actual life capabilities of the user [68]. Another study set up a “milestone” function so that participants could review their goals and thus make adjustments to meet their monthly goals. For example, if a participant sets a fasting glucose target of 60 to 99 mg/dL, a pop-up window displays achievement when the target is reached. Participants can also view weekly or month-long bar and line charts to compare actual versus expected values at different time points and actual versus ideal behavior in order to adjust desired goals as needed [62].

#### Goal Setting Assisted by a Web-Based Coach (n=4)

Four studies [57,58,70,71] involved health coaches assisting participants to help users set specific, measurable, achievable, and realistic goals. Alternatively, in one study, the intervention group was instructed to set specific, measurable, realistic, achievable, and timely goals by selecting small but meaningful changes to fit into their daily lives, which were constantly reviewed and revised during subsequent follow-up [71].

#### Autonomous Goal Tracking and Self-Regulation (n=5)

In addition, goal setting can be done through personal records and individual settings [54,56,69,73,75]. In one study, users were guided through goal-setting modules on weight loss and food choice [75]. In another study, individuals were able to set step goals within an app [56].

### Environmental Background and Resources

#### Overview

Twelve studies created empowering prevention resources for participants by providing access to digital tracking tools and wearable devices [52,53,55,56,61,65,66,69,71,73,74,76]. These devices fall into 3 main categories as follows.

#### Weight Monitoring Equipment (n=7)

Seven studies equipped participants with wireless or Internet-connected scales to automatically track weight changes and transfer data to a database or personal dashboard [52,55,66,69,71,74,76].

#### Motion Monitoring Equipment (n=11)

Exercise monitoring equipment was the most widely used form of resources, with a total of 11 items. Including a pedometer, wrist wear activity tracker, and other intelligent wearable devices [52,53,55,56,65,69,71,73,74,76]. One study [55] allowed participants to use their own wearable devices (eg, Fitbit and Apple Watch).

#### Other Health Monitoring Tools (n=2)

In addition to weight and exercise tracking, 2 studies combined more integrated digital health tools such as continuous glucose monitors, which track blood glucose fluctuations in real time, and heart rate monitors [61]. Alternatively, in the study by Ng et al [71], a glucose meter was provided for self-blood glucose monitoring, and data on the glucose meter and tracker were gradually tracked and synchronized into the application.

### Social Influence

A total of 15 studies identified social influence as a key mechanistic pathway through which eHL facilitated diabetes prevention behaviors [51,53,55,57-59,62,64,66,67,69,71,72,74,76]. The forms can be divided into 2 types. One group used closed digital communities, with 12 studies interacting through web-based groups, private social forums, private chat groups, third-party messaging tools, where participants posted updates, responded to comments, shared lifestyle change experiences, and received feedback from wellness coaches or other members to get online peer support [51,53,55,57,58,62,64,66,67,69,71,74]. For example, in one study, the intervention group was invited to join a peer support chat group hosted by WhatsApp. They could ask questions related to prediabetes and exchange experiences on lifestyle modification, including best practices, challenges faced, and ways to overcome, throughout the study period [71]. In addition to digital communities, 3 studies used structured online discussion activities to enhance interactivity and interaction during continuous live lectures [59,72,76]. For example, in one study, group members explored interactively around each member’s individual case and provided integration opportunities to advise each other based on feedback or their own experience [76].

### Behavioral Regulation

Of the included studies, 16 studies included behavioral regulatory mechanisms as an important component of promoting lifestyle change [50,52,53,56-60,62,63,66,67,70-72,75].

Thirteen studies asked participants to regularly self-record health-related behaviors such as body weight, dietary intake, physical activity, and sleep [50,52,56,59,60,62,63,66,67,70-72,75]. Among them, one high-quality study used the Artificial Intelligence Health Coaching (HCAI) application to prompt users to enter their weight weekly and record the intake of meals and snacks. The system automatically classifies foods as “healthy,” “unhealthy,” or “neutral” to help users form a clearer perception of diet [75]. Another high-quality study introduced the “SPACE” model, which encouraged participants to record five types of daily health-related habits—Sleep, Presence, Activity, Creativity, and Eating. To enhance overall self-awareness [52].

Three other studies used photo-capable “food diaries” in addition to self-recording measures, in which participants were asked to upload photos of each meal, snack, or drink to an App. Health coaches reviewed the records weekly and provided personalized feedback, thereby reinforcing behaviors [53,57,58].

### Beliefs About Competence

Only one study addressed the mechanistic pathway of beliefs about capabilities in diabetes prevention [53]. Based on the concept of social cognitive theory, the Transform program aims to help participants improve self-efficacy, enhance participation, and influence behavior.

### Reinforcement

#### Overview

Among the included studies, 10 reported using reinforcement strategies to enhance participants’ self-efficacy and support the maintenance of health behaviors. These strategies included structured encouragement, coach-delivered encouragement, and incentive-based approaches such as points-based rewards and gamification [50,59,62,64-68,76,77].

#### Systematic Encouragement (n=4)

Four studies used programmed systematic incentive strategies to continuously encourage and strengthen participants’ participation through algorithmically driven feedback [50,64,65,76]. For example, users can see progress and send encouragement notifications. Users are provided with daily and weekly summaries, with weekly summaries graphically providing the amount consumed, total calories consumed, calories burned, and projected weight loss goals for the week for each food group [50].

#### Coach Encouragement (n=4)

Four studies emphasized reinforcement strategies at the interpersonal level, which continuously strengthened participants’ competency beliefs through encouragement of structured programs by health coaches [66], daily information encouragement [67], progress encouragement [77], and social support [59]. In a high-quality study, participants received daily messages from their coaches, such as “No pains, no gains. Focus on the application as much as possible. Record as much as you can, as this gives your coach a sense of your health habits and provides background information for all discussions. The longer you use the app, the more likely you are to stick to your health goals” [67].

#### Points and Gamification Encouragement (n=2)

In one study, participants earned points for setting and achieving goals, which were redeemed for small cash rewards to encourage them to continue reporting health behaviors and achieving goals [68]. Another study added a digital gold reward system to the group chat system, where participants could collect digital currency by completing questionnaires, quizzes, or meeting goals and redeem it for actual prizes. Researchers have further embedded gamification elements to improve participants’ participation in prevention programs [62].

### Memory, Attention, and Decision Processes

Two studies referred to studies related to memory, attention, and decision processes [52,65]. One study helps participants to maintain focus and self-regulation by providing behavioral strategies of mindfulness and decompression [65]. In addition, it predicts the energy and willpower of the individual for a future day. Help individuals understand and control the factors that affect their health behaviors and strengthen their decision-making awareness [52].

### Emotions

Only one study incorporated emotion-related mechanisms in prevention by using artificial intelligence (AI)–enabled guidance to provide compassionate, feedback-informed support. Providing strategic help and emotional support, AI incorporates interactive elements such as reflection, legitimation, respect, support, and partnership to make users feel valued [50].

### The Dimensions of eHL and Intervention Outcomes

The classical framework [26] divides eHL into 6 dimensions. Later, Paige et al [27] proposed a new classification after 2018, which emphasized the interactive use, communication, and behavioral transformation of digital health information in real situations.

### Traditional Literacy

Five studies addressed traditional literacy [51,54,63,65,69]. Four of these directly intervened in participants’ reading comprehension through a forced reading task. For example, in the study by Block et al [65], participants were required to read 2-weekly health notes written by certified diabetes educators and behavioral specialists. In addition, one study conducted an adaptive intervention at the language and cultural level according to the traditional literacy level of participants [51]. The study adapted the DPP curriculum for low-income people, including rewriting content at the fourth and fifth grade reading level, cultural adjustments, and Spanish translation, and adding bilingual and biculture-based health coaches in English and Spanish to help them better understand prevention programs.

### Health Literacy

A total of 27 studies addressed health literacy [50-60,62-77], and interventions were broadly divided into 2 aspects.

First, most studies used health education courses as the intervention core (n=16) [50,51,53,55,57-59,63,64,66,67,69,74-77], covering topics such as healthy diet [54,62,63,65,71], physical activity [54,62,63,65], knowledge of prediabetes [71], etc. Three studies integrated the official Centers for Disease Control and Prevention (CDC)-DPP curriculum.

Second, 7 studies incorporated personalized content into health literacy interventions [6,8,12,19,20,26,27]. In particular, 2 of these studies introduced AI and algorithm-driven personalized health content. The research of Stein and Brooks [75] uses AI to simulate health coaches to provide counseling support, provide strategic help and emotional support, and AI integrates interactive elements such as reflection, legitimization, respect, support, and partnership to make users feel valued. And provide personalized content. The research of Everett et al [68] uses the advanced Sweetch algorithm to provide users with personalized, contextualized, immediate, and timely recommendations to guide users to achieve recommended activity, weight loss, and diet goals in a way that conforms to users’ actual living habits. Personalized goals are constantly adjusted according to the actual behavior of the user to best fit the actual life capabilities of the user. Notifications constitute “teachable moments” that can bring about sustained behavioral change.

### Information Literacy

A total of 20 studies addressed information literacy [50,52-54,56-58,60,62-67,69-72,75,76]. Among them, information recording is the most common type, and participants in 11 studies actively record personal health data such as weight, diet, step count, physical activity, sleep, etc, through apps, dashboards, or online platforms [52,56,60,63,66,67,69,70,72,75,76]. Of these, 4 studies specifically used “health diaries” to enhance information literacy through daily journaling [50,53,62,71]. For example, the study by Srivastava et al [50] entered participants’ food eaten, exercise completed, educational sessions completed, and body weight into a diary, which resulted in automated daily and weekly performance summaries and performance-based tailored daily and weekly recommendations.

Second, information was understood and evaluated through quizzes. In 11 studies, participants were required to complete a course quiz or answer questions [50,53,54,57,58,60,62,64,65,69,71]. For example, in the study by Fitzpatrick et al [58], participants were required to complete questionnaires, quizzes, and open-ended questions. Course completion was defined as completion of the quiz attached to the end of the course content.

### Science Literacy

Among the included studies, 9 studies involved the cultivation of scientific literacy [50,52,56,61-63,71,75,76]. Of these, 7 studies focused on dietary knowledge based on scientific principles [50,56,61,63,71,75,76]. For example, in the study of Stein and Brooks [75], the AI-based text interactive HCAI system will classify the food entered by the user into 3 categories of “healthy,” “unhealthy,” and “neutral” to facilitate the user to establish a food recognition mechanism. In the study by Ng et al [71], the application module covered multiple aspects of healthy eating and practical nutrition advice for people with prediabetes, including content on energy balance, portion control, healthy eating based on Malaysian indigenous foods, glycemic index, dietary fiber intake, fat and protein food types, and alcohol intake.

In addition, 2 studies addressed scientific mechanisms of disease [62,71]. For example, in the study of Chung et al [62], the TCM mHealth app included knowledge about constitution classification, meridian energy, and descriptive illustrations and video teaching of Qigong types such as Baduanjin and abdominal breathing. The Ng et al [71] study provided knowledge on the pathophysiology and complications of prediabetes, health benefits, and specific recommendations of physical activity, health risks of smoking, stress management strategies, and motivation training for lifestyle changes in the app. These science literacy training strategies help participants to understand the causal relationship between health behaviors and disease prevention from a scientific perspective, thereby improving health decision-making ability.

### Media Literacy

Of the 28 included studies, none explicitly addressed the cultivation of media literacy, which is the ability to critically evaluate digital health information sources, identify misleading content, or understand algorithmic mechanisms, among other things.

### Computer Literacy

According to the analysis of the included studies, 11 studies involved the cultivation of computer literacy [52,53,56-58,62,63,67,70,71,77]. These interventions mainly help participants master the skills of using digital health tools through platform operation training and multimedia content creation and uploading.

First, platform operation training is the basis of computer literacy training [52,56,62,63,67,70,71,77]. For example, in the study by Lim et al [56], participants in the intervention group were introduced to the nBuddy Diabetes mobile app. They were asked to download the nBuddy Diabetes app and receive training, and in the study by Michaelides et al [67] and Toro-Ramos et al [70], participants were asked to learn how to use the Noom app and interact online with a health coach.

Second, 3 studies involved multimedia content creation and uploading. For example, participants were asked to track eating behavior by taking and uploading eating photos [53,57,58].

### Functional eHealth Literacy

Of the included studies, 4 studies addressed functional eHL [51,54,63,65].

First, the ability to read health information is a core competency of functional literacy [54,63,65]. For example, in the study by Vadheim et al [63], each participant was asked to read books that estimated the fat and calorie content of specific foods and to correctly record fat, body weight, calories, and minutes of activity in an electronic diary. The study by Islam et al [54] designed practice activities in workshops to help participants master important nutrition-related skills, such as reading food nutrition labels.

Second, culturally adaptive content adjustment reflects the attention paid to people with different levels of functional literacy [51]. In the study by Kim et al [51], the DPP curriculum was adapted for low-income populations, including rewriting content to fourth- and fifth-grade reading levels, cultural adjustment and Spanish translation, and adding bilingual and bicultural health coaches in English and Spanish. This design lowers the reading threshold and ensures that participants of different educational backgrounds and language abilities can effectively understand and use digital health information.

### Communicative eHealth Literacy

According to the analysis of the included studies, 20 studies related to communicative eHL [51,53-59,64-67,69-76].

A total of 11 studies demonstrated the cultivation of participants’ collaboration ability [51,54,57-59,66,69,71,73,74,76]. These studies promote participant prevention through discussion, providing social support, sharing experiences, etc. For example, in the study of Islam et al [54], the study set up a “brainstorming” session, where peer leaders shared questions/prompts related to healthy eating, physical activity, and healthy lifestyle barriers (stress, difficult emotions, school/work schedule, family stress, etc), and participants were invited to share ideas. In the study by Collins et al [59], program participants were allowed to interact during live lectures to share successes/challenges and provide support to each other.

### Translational eHealth Literacy

Of the included studies, 12 involved translational eHL [50,52-54,56,61,62,65,68,70,71,76]. The common feature of these studies is the immediate translation of digital health information and self-monitoring data into concrete, executable behavioral goals through regular individualized feedback and the dynamic revision of these goals in subsequent stages. For example, in the study of Block et al [65], the system generates personalized dietary and exercise goals for participants every week and automatically adjusts small step goals for the next week based on their actual achievement in the previous week. Participants are required to report their goal completion every week, and the system continuously updates the follow-up plan accordingly. In contrast, in the study by Alwashmi et al [53], this regulatory process was mainly accomplished by human coaches. The coaches reviewed participants’ follow-up weekly, provided specific recommendations for diet and meal plan changes, and helped participants set more feasible goals for the coming week, thereby reinforcing behavior change on the basis of progress.

### Critical eHealth Literacy

Among the 28 studies included, none addressed critical eHL, which is the ability to evaluate the credibility, relevance, and risks of health information shared and received online.

### Intervention Outcomes

#### Engagement

Overall, studies reported high levels of participant engagement [50,52,55,58-60,63-65,68,69,74-77]. Of note, 2 studies achieved 100% participation [52,76]. In one of these studies, app+ completion rates reached 100% in the intervention group. Participants used app+ for an average of 37 days over 12 weeks [52]. In the other study, meeting attendance was 100%, participants had a mean age of 65.4 years, wore a fitness tracker daily, were weighed more than 3 times per week, and recorded food intake more than 3 times per week. During the 6-month reporting period, participants adhered to the target calorie range 74% of the time. The initial daily step goal was 5000, and 92% of participants maintained this goal through 6 months [76].

In terms of course completion, participation was better [50,58,59,63,69,77]. Among them, in one study, 88% of participants completed 16 weeks of courses [63]. In another study, 95% completed at least 4 weekly education courses, and 92% completed 9 or more courses [69]. Across different educational stages, nearly 65% of high school or equivalent people completed the first stage of courses. For participants who graduated from college, the completion rate of phase 1 reached 75.5% [59].

In terms of online platform use, participants similarly demonstrated strong engagement. The study showed that all participants completed at least 4 core modules, and participants logged in to the online intervention 76 times on average, weighed 89 times, sent 46.5 group messages, and sent 20.5 messages to the health coach [64]. Another study reported that 70.8% of intervention group participants interacted with the program at least once a week. After 6 months, 70.6% were still actively using the Alive-PD system [65]. In another study, participants logged on an average of 22.5 times weekly, weighed themselves 7.3 times weekly, performed 11 diet/activity tracking sessions, and had 1 private message with a health coach. In addition, 96% (n=455) of participants were enrolled in the digital DPP program for the full 12 months [58].

#### Acceptance or Satisfaction

Six of the included studies [52,54,60,64,68,75] explored acceptance or satisfaction. Six studies received positive reviews from participants, and in 2 of these studies, participants clearly expressed a preference for prevention programs [52,60]. In the study by Moin et al [64], women veterans with prediabetes after the prevention program rated the program as a good fit with perceived health needs, “it’s perfect,” “it can keep its own pace,” “flexible, it can be free from time constraints,” the program was integrated into daily life and made people feel responsible. In the study of Everett et al [68], Sweetch mobile platform had a high acceptance among participants. Most participants agreed or strongly agreed that they would like to use the Sweetch app often (74%), thought the app was easy to use (83%), thought the features were well integrated (72%), thought most people could learn to use the app quickly (89%), and felt confident in using the app (77%). The value and practicability of the project were highly recognized by the participants. In the study by Islam et al [54], participants indicated that the online adolescent health education program was beneficial and applicable to their lives, giving them a deeper understanding of healthy eating and an active lifestyle. When asked to describe items with adjectives, the most commonly used words were “interesting” and “useful.” Participants appreciated the Let’s Move activities and discussed how the activities had helped them understand healthy eating and share what they had learned with friends and family. Most participants found the game both fun and rewarding, enjoyed teamwork, enjoyed competition, felt that the game helped participants become more comfortable with each other, and appreciated that the game challenged them to remember important concepts they had learned.

In addition, 3 studies [60,64,72] also pointed out the challenges of eHL participation in prevention. In the study by Moin et al [64], participants expressed an aversion to records, a lack of openness online, and a lack of computer literacy, leading to comprehension difficulties.

In the study of Yeh et al [60], computer literacy also encountered challenges. Some participants experienced difficulties in the first 2 weeks, including difficulty recording food intake and low Facebook group participation, with only 2 people interacting. Participants had barriers to software use. In the study by Audet et al [72], older participants still complained about the use of video technology in group meetings and expressed difficulties in developing computer literacy.

In addition, the study of Yeh et al [60] also pointed out the challenges brought by cultural differences. Chinese-American participants expressed challenges in recording weight in prevention programs. Because in Chinese culture, meals are usually shared in a family format, and there are no individual portion sizes.

#### Body Weight

Of the 28 included studies, 22 reported on weight management results, showing a significant effect of eHL in promoting weight management in people at risk of diabetes [50,51,53,55-61,63-70,74-77].

In short-term intervention studies (≤6 mo) [50,53,56,61,63-65,68,75-77], weight loss has been substantial. Of these, 6 studies explicitly reported that participants lost 5% or more of their body weight [50,53,63-65,76]. For example, in the study by Srivastava et al [50], all 6 users who completed the study achieved the CDC target of 5% weight loss at 6 months, with an average weight loss of approximately 9.0%. In another high-quality study [76], weight was reduced by 5.3% at 3 months and 8.4% at 6 months in the intervention group, with 90% of participants achieving and maintaining the target weight loss of 5% or more and a reduction in BMI from baseline 31.3 to 29.06.

Weight loss was maintained in medium- and long-term intervention (≥9 mo) studies. Five studies [55,57,59,67,70] reported weight changes in participants from 9 months to 65 weeks later. For example, Katula et al [55] showed that patients in the d-DPP group lost an average of 5.49% of their initial weight at 12 months and were 61% more likely to have clinically significant weight loss (≥5%) than those in the control group, and that greater participation was associated with greater weight loss, especially among participants 65 years of age or older.

Six studies reported trends in weight change at different time points [51,58,60,66,69,74]. For example, Castro Sweet et al [69] showed that participants lost an average of 6.5% of body weight at 16 weeks, increased to 8.0% at 6 months, and remained at 7.5% at 12 months, with an average weight loss of 13‐14 pounds. Long-term follow-up by Sepah et al [74] showed a mean weight loss of 4.9% after 1 year and 4.3% after 2 years among patients who completed the program, demonstrating sustained weight loss maintenance. Of note, in the study by Fitzpatrick et al [58], the average weight loss was 8.6 pounds (4.0%) within 12 months, which was clinically significant, but at 24 months, the percentage of weight loss was 5.7 pounds, which was no longer statistically significant, and long-term weight maintenance was still a challenge.

#### Diabetes Indicators

Of the 28 included studies, 12 reported improvements in measures of glycemic control [51,55,56,59,61,62,65,66,68-70,74]. Ten of these studies showed positive results [51,55,56,59,61,62,65,66,69,74], and 2 studies [68,70] reported no significant change from baseline in fasting plasma glucose and glycosylated hemoglobin (HbA_1c_).

Seven studies reported reductions in HbA_1c_ [51,55,59,62,66,69,74]. Among them, 3 studies [62,66,69] found sustained and sustained reductions in HbA_1c_ over time after eHL engagement prevention studies. For example, in the study by Sepah et al [74], the mean reduction in glycated hemoglobin levels was 0.38% after one year and 0.43% after 2 years. In a long-term study by Sepah et al [66], significant reductions in glycated hemoglobin levels were observed at week 16 and at years 1, 2, and 3.

In addition, 5 studies reported improvements in fasting plasma glucose [56,61,62,65,69]. In a high-quality study, participants’ fasting blood glucose decreased by 0.41 at 6 months, and their Framingham 8-year risk for diabetes decreased from 16% to 11%. Most encouragingly, 40.5% of the intervention group achieved normal fasting glucose levels at 6 months.

In terms of other diabetes-related measures, one study showed significant within-group improvements in systolic and diastolic blood pressure, total cholesterol, and low-density lipoprotein cholesterol after 6 months in the intervention group [56], and another study combined a TCM mobile health app intervention. It was shown that participants performed better than the control group in terms of body constitution (improvement of Yang deficiency and sputum stasis), body energy improvement, and health-related quality of life (PCS and MCS) after 4 months. Prevention that demonstrates eHL engagement appears to be more likely to improve physical energy and health-related quality of life [62].

#### Behavioral Change

Results of a total of 13 studies showed that eHL participation in prevention research promoted the improvement of health behaviors of participants [50,52-54,56,61,65,68,72,73,75,77]. Of these, 8 studies involved improvements in dietary behavior among participants [52,54,56,61,65,72,75,77]. For example, in the study by Lim et al [56], the daily intake of total energy, carbohydrates, total fat, saturated fat, and sugar decreased significantly after 6 months in the intervention group. Another pilot study [54] found that participants learned about healthy eating during the program and were able to share what they learned with family and friends. As one participant commented, “The program helped me to adjust my eating habits, to know what to eat, how much to eat, and how much to plan.”

Second, 9 studies showed changes in physical activity among participants [50,52,53,57,61,65,68,72,73]. For example, in the study by Ekezie et al [73], participants significantly increased their physical activity levels and average daily steps. A wrist-worn activity monitor linked to a digital app tracked daily steps in real time, and the “physical activity challenge” feature of the program was considered a key enabler of goal attainment, helping participants stay focused and motivated to continue achieving their goals over a longer period of time, they said. Another study further validated the improvement in motor behavior. Zahedani et al [61] found that participants with prediabetes had a significant increase in their daily physical activity after 3 months of intervention, with the average duration of physical activity approximately doubling. As an objective measure, the mean daily heart rate rose by 6.3 minutes/day.

#### Self-Efficacy

In addition, 2 studies also reported significant improvements in self-efficacy among participants [59,65]. In the study by Collins et al [59], participants showed statistically significant improvements in the ability to perform 18 self-reported health promotion behaviors.

## Discussion

### Principal Findings

To the best of our knowledge, this is the first systematic literature review of T2D prevention in at-risk populations, which aims to deeply explore the mechanism of eHL in T2D prevention in at-risk populations and the participation and effects of multidimensional prevention. Our core finding is that eHL, although extremely uneven in its application, still achieves significant preventive effects. This reveals systemic problems in current prevention research and future directions for improvement.

In this review, we mapped intervention components and reported implementation strategies from the included studies to the TDF domains and quantified how frequently each domain was reflected across the 28 studies. The use of these pathways is extremely uneven. Almost all studies relied on the “knowledge” approach (28/28, 100%), while behavioral regulation (16/28, 57.1%) and social influence (15/28, 53.6%) were also widely used. However, few studies involved emotions (1/28, 3.6%), beliefs about competence (1/28, 3.6%), memory, attention, and decision processes (2/28, 7.1%). This implies that current research constructs a “surface” rather than a “deep” prevention model, with much focus on knowledge that is easily transmitted and behaviors that are easily monitored, but the potential to stimulate intrinsic motivation for change has been largely neglected.

In all the studies included in this review, health literacy was central in terms of understanding. The original intention of such prevention is consistent with the CDC in the United States, which is to translate scientific knowledge into action to improve public health [78]. In addition, 18 studies showed a trend toward more standardized prevention content based on DPP content. Similarly, behavioral regulation and social incentives are relatively easy to achieve through regular recording with various types of incentives. Perhaps the reason for the widespread adoption of easy-to-implement mechanisms lies in their operability. The convenience of such preventive measures may obscure a fundamental question: Is the easy-to-implement path the most long-lasting mechanism?

Our findings suggest that, in terms of implementation, the included studies focused primarily on the goals of algorithmic systems and digital coaches assisting design, and that this design difference has theoretical and practical implications for self-determination theory. Self-determination theory emphasizes autonomy as a key intrinsic motivation for promoting behavior change [79]. Overreliance on algorithms or digital coaches may undermine participant autonomy. Second, the emotional and psychological pathways are also neglected. This is consistent with previous findings that psychological factors have been underreported in digital health care interventions [80]. For example, negative emotions such as the frustration caused by weight loss and blood glucose rebound were obviously ignored in the research, and negative emotions are often one of the reasons for interrupting self-control behavior [81]. Such neglect tends to lead to long-term prevention failure.

Second, eHL is by no means a trivial set of skills in a highly digital environment. Rather, it combines knowledge and skills from a variety of fields [82]. Our review found that the framework of Norman et al [26] and Paige et al [27] is highly fragmented and selectively applied in practice.

Specifically, health literacy and information literacy were the 2 most frequently involved dimensions. On the contrary, although the attention to scientific literacy increased year by year, the attention to traditional literacy was completely absent after 2022. Media literacy is more worrisome, and none of the prevention studies addressed the cultivation of media literacy.

There are many explanations for the uneven distribution of dimension involvement in prevention. First, the concept of eHL has been discussed and applied in the field of public health and health communication since it was proposed by Norman et al [26]. In contrast, media literacy stems more from the field of media studies and has not yet been fully integrated. This has led to a critical gap: the Internet has become an important channel for the dissemination of health information for the prevention of T2D, but the requirements of media literacy, such as how to identify the correct medical information, have not been systematically incorporated into preventive medical interventions.

However, the interest in scientific literacy has been increasing in recent years, especially after the COVID-19 pandemic, which is a positive sign to some extent. However, it may simply be because of the external pressure of the global nature of the pandemic.

In addition, the lack of traditional literacy, perhaps due to the popularity of digitalization, researchers have neglected that online functions still require basic reading and writing skills. It has been found that a disadvantage in written and oral language skills can be a barrier to accessing web-based health information [83]. This means that current prevention studies may be inadvertently excluding participants who have low literacy and difficulty reading and typing. People with lower education levels are generally at higher risk of chronic diseases [84]. Thus, the lack of traditional literacy is not only a study design issue, but also a health equity issue. To ignore traditional literacy is to exclude the most vulnerable from digital health help, exacerbating divisions.

In 2018, Paige et al [27] classified the new model as a further improvement of the old model, from “instrumental skills” to “interactive application ability.” However, this study found further “selective neglect” in the study: communicative eHL and translational eHL were more involved in prevention, but functional eHL was less involved, and critical eHL was completely missing. Once again, this demonstrates the “selective” application mechanism of eHL in practical prevention research.

Encouragingly, research has begun to directly acknowledge and respond to inequalities in functional literacy. The diabetes prevention curriculum was adapted for low-income people, rewritten to fourth to fifth grade reading difficulty, culturally adjusted, and translated into Spanish, and added bilingual and bicultural health coaches. The breakthrough of this approach is that it takes the initiative to consider and lower the threshold of health information, so that participants with different cultural backgrounds and language abilities have the opportunity to participate in digital health, which is particularly critical in the field of diabetes prevention. Because the lack of functional eHL can further lead to the emergence of a digital divide, there are significant differences in the need for technical training among different populations. Among the included studies, studies involving, for example, older adults [58,76], their training in the use of digital tools need more targeted attention.

Second, communicative eHL had the highest frequency, also responding to the absence of the “emotional information factor” in the path effect. For those at risk, the experience of being “understood and supported” is itself a source of motivation to continue to participate. This motivation is further strengthened by the translational eHealth hormone. Participants were trained and guided repeatedly, leading to more long-term, routine behavior change. An emerging trend observed in this study is that AI is gradually becoming an important tool for eHL interventions. In one of the included studies, an AI health guidance application (HCAI application) provided a human-like guidance experience and personalized content [75]. AI is playing an increasingly important role in enhancing diabetes prevention and management [85]. The introduction of AI, to some extent, strengthens “translational eHealth literacy,” and AI shows great potential in long-term prevention.

The absence of critical eHL, however, is consistent with media literacy. This is also one of the biggest concerns of this review. The current digital environment is extremely complex, and it is very important for individuals to be able to include and judge the credibility and reliability of information sources. The included studies were mainly conducted within the framework of guidance from medical professionals, and participants were not required to assess whether such programs were disinformative. However, once this support is withdrawn, and the participants’ lack of autonomy is fostered, it is extremely easy to lead to difficulties in self-management. Therefore, we suggest that future prevention research could embed training modules on “how to avoid false judgments” while maintaining “learning to make good decisions,” for example, scoring the credibility of health information, exercises on checking health information, etc, mitigating the double risks of information overload and misinformation.

More urgently, the review 5 years ago already noted that while health literacy was the most assessed domain, none of the studies evaluated all 6 domains of the eHL model [86]. The results of this review still show that none of the 28 studies designed and evaluated eHL as a comprehensive, unified concept. Although the path application of eHL was not balanced with the dimensions of participation, our review suggests that eHL is capable of achieving positive outcomes in terms of T2D prevention outcomes for at-risk populations. The cultivation of eHL can improve the participation, satisfaction, weight management, diabetes-related physiological indicators, health behavior change, and self-efficacy of the prevention of T2D in the risk population. This finding is consistent with existing results on the impact of eHL on health behaviors [87-92].

Therefore, the importance and effectiveness of eHL in the prevention of T2D in the at-risk population are clear. But our findings need to highlight how eHL can be a more sustainable long-term driver that needs to be addressed by health communication. In the future of T2D prevention, especially with the rapid development of AI, there is an urgent need to incorporate “autonomous” literacy training without professional guidance into prevention design, rather than continue to assume that participants will automatically have these abilities. Only in this way can eHL help public health mitigate the imbalances brought about by the information age, especially in at-risk and vulnerable populations, and reduce the incidence of T2D in the long term.

This review is novel in providing a theory-driven, mechanistic synthesis of how eHL supports T2D prevention in at-risk populations. We mapped and quantified intervention components and implementation strategies against the TDF and multidimensional eHL frameworks, revealing a marked imbalance in mechanisms: studies largely rely on knowledge and easily operationalized behavioral pathways, while underrepresenting mechanisms critical for long-term maintenance (eg, emotions, support toward autonomy/self-efficacy, and cognitive decision processes). Our contribution is a practical mechanism-by-dimension framework and an evidence-gap agenda, highlighting common omissions such as critical eHL and media literacy, and limited attention to functional literacy and access barriers as determinants of equity. In real-world implementation, future digital/AI-enabled prevention should move beyond information delivery to embed affective and autonomy-supportive design, misinformation-resilience (critical appraisal) training, and accessibility adaptations for vulnerable groups; AI should be integrated cautiously to avoid undermining autonomy and to enhance long-term sustainability and health equity.

### Limitations

This review has several limitations. First, although the study aimed to explore the impact of eHL on the prevention of T2D, we did not conduct a meta-analysis due to the substantial heterogeneity observed among the included studies in terms of research design, intervention approaches, and outcome measures.

Second, as “eHealth literacy” remains an emerging concept in the research landscape, the volume of available literature is still limited. Consequently, the relatively small sample of studies included in this review may restrict the comprehensiveness of our understanding of the field.

Third, the thematic analysis used in this review was qualitative in nature. Most of the included studies did not directly investigate the impact of eHL on T2D prevention but instead provided indirect evidence or descriptions of relevant interventions. As a result, the mechanisms through which different dimensions of eHL contribute to the prevention process were derived primarily through interpretive synthesis rather than quantitative validation. This type of analysis may be subject to researcher subjectivity and carries a risk of reporting bias. To mitigate this risk and enhance the transparency of the findings, all authors were involved in reviewing each stage of the data analysis process.

Accordingly, several strategies are proposed for future research. First, to address the high degree of heterogeneity across studies, future research should consider conducting more randomized controlled trials with clearly defined control groups and standardized intervention protocols. Such studies would help generate high-quality empirical data suitable for quantitative synthesis and lay the groundwork for future meta-analyses.

Second, future studies should conceptualize eHL as an integrated and unified construct rather than examining its dimensions in isolation. Researchers are encouraged to adopt standardized measurement tools, such as the eHL Scale, for systematic assessment. Moreover, rather than focusing solely on the indirect role of eHL in digital interventions, future studies should investigate its direct effects as a core variable. Doing so will help clarify its independent contributions and support the standardization and theoretical development of eHL in the context of disease prevention.

Third, to reduce potential bias and enhance the credibility and validity of findings, future research may consider incorporating external peer review or expert consensus methods in the analytical process. For example, using the Delphi method to collect structured input from multidisciplinary experts can enhance the robustness and transparency of the results. These strategies help ensure that the conclusions are not solely reliant on the subjective judgments of the primary research team.

### Conclusions

This mechanistic systematic review integrated the TDF and eHL models through theory-driven thematic analysis. It focuses on the pathways through which eHL influences prevention among individuals at risk for T2D. Findings indicate that eHL supports sustained engagement and self-management through mechanisms such as knowledge and skill acquisition, goal setting, behavioral regulation and self-monitoring, resource usage, and social influence, and is associated with outcomes including weight control, improved glycemic indicators, self-efficacy, and lifestyle change. Most studies reported high engagement rates and satisfaction levels, suggesting good acceptability and feasibility of intervention programs.

However, the exploration of mechanisms for T2D prevention across current studies remains uneven. Emotional support, beliefs about competence, and autonomy were underaddressed, while critical eHL and media literacy dimensions were largely absent. The default assumption of traditional and functional eHL’s importance may inadvertently exclude resource-limited, at-risk populations, exacerbating health inequities and the digital divide.

Future digital DPPs must better serve diverse populations. Our findings reveal that most programs focus heavily on information delivery but neglect critical factors that help people sustain behavior change: managing emotional setbacks, supporting personal autonomy, and distinguishing reliable health information from misinformation. We recommend that programs incorporate emotional support to address frustration and disappointment, empower users to make independent choices rather than relying solely on algorithm recommendations, and teach simple skills for evaluating web-based health information credibility. Additionally, programs should include media literacy training, use engagement mechanisms such as incentives and encouragement, and adapt designs to accommodate different ages, education levels, and digital access capabilities. Only through such comprehensive, equity-focused approaches can eHL serve as an effective public health tool for broader populations, particularly vulnerable and at-risk groups.

## Supplementary material

10.2196/77788 Multimedia Appendix 1 Searching strategies.

10.2196/77788 Checklist 1 PRISMA 2020 checklist.

10.2196/77788 Checklist 2 PRISMA-S checklist.

## Abbreviations

AI: artificial intelligence
CDC: Centers for Disease Control and Prevention
DPP: Diabetes Prevention Program
eHL: eHealth literacy
HbA_1c_: glycosylated hemoglobin
HCAI: Artificial Intelligence Health Coaching
MMAT: Mixed Methods Appraisal Tool
PRISMA: Preferred Reporting Items for Systematic Reviews and Meta-Analyses
PRISMA-S: Preferred Reporting Items for Systematic Reviews and Meta-Analyses–Search
RQ: research question
T2D: type 2 diabetes
TDF: Theoretical Domains Framework
WOS: Web of Science
