# Supplementary material for: eHealth Literacy and Type 2 Diabetes Prevention Among At-Risk Populations: Mechanistic Systematic Review Using Theory-Driven Thematic Analysis
Source: J Med Internet Res. 2026 Mar 10;28:e77788. doi: 10.2196/77788 (PMC12975002; doi:10.2196/77788)
Supplement: Multimedia Appendix 1 [file jmir-v28-e77788-s001.docx]

| **Appendix 1. Searching strategies** | | |
| --- | --- | --- |
| Scopus | TITLE-ABS-KEY ( ( "eHealth" OR "e-health" OR "mHealth" OR "m-health" OR "mobile health" OR "digital health" OR "internet health" OR "telehealth" OR "telemedic*" OR "computer health" OR "computer-based health" OR "web health" OR "web-based health" OR "web based health" OR "online health" OR "online-based health" OR "online based health" OR "health" OR "media" OR "computer" OR "virtual" OR "intelligent" OR "technolog*" OR "Web 2.0" OR "web based" OR "online based" OR "phone*" OR "app*" )  AND ( "literac*" OR "comprehension" OR "skill*" OR "ability" OR "knowledge" OR "seek*"OR "find*" OR "search*" OR "understand*" OR "apprais*" OR "evaluat*" OR "assess*" OR "access*" OR "apply" OR "use*" OR "communication" OR "interaction" OR "efficacy" OR "engagement" )  AND ( "diabetes prevent*" OR "diabetes prevention" OR "type 2 diabetes prevent*" OR "type II diabetes prevent*" OR "T2D prevent*" OR "T2DM prevent*" OR "prediabetes prevent*" OR "non-insulin-dependent diabetes mellitus prevent*" OR "adult-onset diabetes prevent*" OR "NIDDM prevent*" )  AND ( "at-risk" OR "high-risk" OR "risk population" OR "prediabetes" OR "overweight" OR "fat" OR "obesity" OR "sedentary behavi*" OR "sedentary lifestyle*" OR "physical inactiv*" OR "lack of exercise" OR "genetics" OR "family history" OR "gestational diabetes" OR "hypertens*" OR "dyslipid*" OR "insulin resistance" OR "acanthosis nigricans" OR "non-alcoholic fatty liver disease" OR "NAFLD" OR "metabolic syndrome" OR "chronic stress" OR "depression" OR "poor sleep" OR "western diet" OR "unhealthy diet" OR "suboptimal diet" ) ) AND PUBYEAR > 1999 AND PUBYEAR < 2026 AND ( LIMIT-TO ( DOCTYPE , "ar" ) ) AND ( LIMIT-TO ( LANGUAGE , "English" ) ) | 2002 |
| Web of science | TS=(("eHealth" OR "e-health" OR "mHealth" OR "m-health" OR "mobile health" OR "digital health" OR "internet health" OR "telehealth" OR "telemedic*" OR "computer health" OR "computer-based health" OR "web health" OR "web-based health" OR "web based health" OR "online health" OR "online-based health" OR "online based health" OR "health" OR "media" OR "computer" OR "virtual" OR "intelligent" OR "technolog*"  OR "Web 2.0" OR "web based" OR "online based" OR "phone*" OR "app*")  AND ("literac*" OR "comprehension" OR "skill*" OR "ability" OR "knowledge" OR "seek*"OR "find*" OR "search*" OR "understand*" OR "apprais*" OR "evaluat*" OR "assess*" OR "access*" OR "apply" OR "use*" OR "communication" OR "interaction" OR "efficacy" OR "engagement" )  AND ("diabetes prevent*" OR "diabetes prevention" OR "type 2 diabetes prevent*" OR "type II diabetes prevent*" OR "T2D prevent*" OR "T2DM prevent*" OR "prediabetes prevent*" OR "non-insulin-dependent diabetes mellitus prevent*" OR "adult-onset diabetes prevent*" OR "NIDDM prevent*")  AND ("at-risk"OR"high-risk" OR "risk population" OR "prediabetes" OR "overweight" OR "fat" OR "obesity" OR "sedentary behavi*" OR "sedentary lifestyle*" OR "physical inactiv*" OR "lack of exercise" OR "genetics" OR"family history"OR "gestational diabetes" OR"hypertens*" OR"dyslipid*" OR "insulin resistance" OR "acanthosis nigricans" OR "non-alcoholic fatty liver disease" OR "NAFLD" OR "metabolic syndrome" OR "chronic stress" OR "depression"OR "poor sleep" OR "western diet" OR "unhealthy diet" OR "suboptimal diet"))  AND PY=2000-2025  AND LA=English  AND DT=Article | 2045 |
| PubMed | (  "eHealth"[Title/Abstract] OR "e-health"[Title/Abstract] OR "mHealth"[Title/Abstract] OR "m-health"[Title/Abstract] OR  "mobile health"[Title/Abstract] OR "digital health"[Title/Abstract] OR "internet health"[Title/Abstract] OR  "telehealth"[Title/Abstract] OR "telemedic*"[Title/Abstract] OR "computer health"[Title/Abstract] OR  "computer-based health"[Title/Abstract] OR "web health"[Title/Abstract] OR "web-based health"[Title/Abstract] OR  "web based health"[Title/Abstract] OR "online health"[Title/Abstract] OR "online-based health"[Title/Abstract] OR  "online based health"[Title/Abstract] OR "health"[Title/Abstract] OR "media"[Title/Abstract] OR  "computer"[Title/Abstract] OR "virtual"[Title/Abstract] OR "intelligent"[Title/Abstract] OR  "technolog*"[Title/Abstract] OR "Web 2.0"[Title/Abstract] OR "web based"[Title/Abstract] OR  "online based"[Title/Abstract] OR "phone*"[Title/Abstract] OR "app*"[Title/Abstract]  )  AND  (  "literacy"[Title/Abstract] OR "comprehension"[Title/Abstract] OR "skill"[Title/Abstract] OR "ability"[Title/Abstract] OR  "knowledge"[Title/Abstract] OR "seek*"[Title/Abstract] OR "find*"[Title/Abstract] OR "search*"[Title/Abstract] OR  "understand*"[Title/Abstract] OR "apprais*"[Title/Abstract] OR "evaluat*"[Title/Abstract] OR  "assess*"[Title/Abstract] OR "access*"[Title/Abstract] OR "apply"[Title/Abstract] OR "use*"[Title/Abstract] OR  "communication"[Title/Abstract] OR "interaction"[Title/Abstract] OR "efficacy"[Title/Abstract] OR  "engagement"[Title/Abstract]  )  AND  (  "diabetes prevent*"[Title/Abstract] OR "diabetes prevention"[Title/Abstract] OR  "type 2 diabetes prevent*"[Title/Abstract] OR "type II diabetes prevent*"[Title/Abstract] OR "T2D prevent*"[Title/Abstract] OR "T2DM prevent*"[Title/Abstract] OR "prediabetes prevent*"[Title/Abstract] OR  "non-insulin-dependent diabetes mellitus prevent*"[Title/Abstract] OR  "adult-onset diabetes prevent*"[Title/Abstract] OR "NIDDM prevent*"[Title/Abstract]  )  AND  (  "at-risk"[Title/Abstract] OR "high-risk"[Title/Abstract] OR "risk population"[Title/Abstract] OR  "prediabetes"[Title/Abstract] OR "overweight"[Title/Abstract] OR "fat"[Title/Abstract] OR "obesity"[Title/Abstract] OR  "sedentary behavi*"[Title/Abstract] OR "sedentary lifestyle*"[Title/Abstract] OR  "physical inactiv*"[Title/Abstract] OR "lack of exercise"[Title/Abstract] OR "genetics"[Title/Abstract] OR  "family history"[Title/Abstract] OR "gestational diabetes"[Title/Abstract] OR "hypertens*"[Title/Abstract] OR  "dyslipid*"[Title/Abstract] OR "insulin resistance"[Title/Abstract] OR "acanthosis nigricans"[Title/Abstract] OR  "non-alcoholic fatty liver disease"[Title/Abstract] OR "NAFLD"[Title/Abstract] OR  "metabolic syndrome"[Title/Abstract] OR "chronic stress"[Title/Abstract] OR "depression"[Title/Abstract] OR  "poor sleep"[Title/Abstract] OR "western diet"[Title/Abstract] OR "unhealthy diet"[Title/Abstract] OR  "suboptimal diet"[Title/Abstract]  )  AND ("2000/01/01"[Date - Publication] : "2025/12/31"[Date - Publication])  AND English[Language] | 1362 |
